# A Good Policy for Guaranteed Safe Practice of Complementary and Alternative Medicine, Usage of Disposable Cupping Cups

**DOI:** 10.1155/2015/970327

**Published:** 2015-06-02

**Authors:** Tae-Hun Kim, Jung Won Kang

**Affiliations:** ^1^Korean Medicine Clinical Trial Center, Korean Medicine Hospital, Kyung Hee University, Seoul 130-872, Republic of Korea; ^2^Department of Acupuncture & Moxibustion, College of Korean Medicine, Kyung Hee University, Seoul 130-872, Republic of Korea

This is a comment on “Addendum: Safety Standards for Gua Sha (Press-Stroking) and Ba Guan (Cupping)” [1]. Interventions of complementary and alternative medicine (CAM) are facing huge challenges whether their effectiveness and safety can be supported by concrete evidence. Although CAM is widely used and takes considerable charge of public health worldwide, benefits and harms related to each CAM intervention are frequently disputable, which urges relevant researchers and academic societies to conduct rigorous clinical trials and to support information for medical decision with evidence-based knowledge through reputable clinical guidelines. One of the major factors that may be inhibiting referrals of CAM treatments by biomedical doctors is the absence of appropriate information related to CAM interventions [1]. In this sense, this current safety guideline on the* Gua sha* (press-stroking) and* Ba guan* (cupping) by Nielsen et al. deserves a passionate welcome, even though it seems to have room for improvement, especially in the field of cupping [2].

Compromising with reality inevitably, recently published addendum of this guideline added a process for disinfection or sterilization of cupping devices, which might retract clinical importance of this guideline [3]. Although recommended sterilization process might be effective in preventing most unnecessary adverse events related to wet-cupping, it seems quite clear that all the blood-borne viral infection cannot be blocked perfectly with it. Single use of cupping device is the best way we need to pursue, and it is not an impossible measure practically. In this letter, we want to show a good example of dissemination of single-use, disposable cupping cup through the government policy in Korea.

Korean National Health Insurance Service has reimbursed the material costs of single-use, disposable cupping cups for wet-cupping treated by doctors of Korean Medicine (KM) abiding by the decision of National Health Insurance Policy Deliberative Committee, Ministry of Health & Welfare Since 2012. Presently, there are seven companies producing disposable cups after obtaining approval from the ministry (Table 1). Any licensed doctors of KM who use these products for wet-cupping can get reimbursement from the service after easy registration in the website of Korean Health Insurance Review & Assessment Service (http://www.hira.or.kr/). From our experience of clinical practice and trials, these single-use cups can be utilized very safely and any specific adverse events hardly occur [4].

Because there is not yet any survey result on the current state of utilization of disposable cupping in Korea, future related study will be necessary on the real usage of disposable cupping cups in KM clinics. But it seems to be that more and more doctors of KM move to using disposable cupping cups because they are safer than reused ones, and doctors do not need to pay for them. Patients who are prejudiced against the wet-cupping therapy for its unsanitary nature will participate in cupping treatment willingly in this situation. As several times used, sterilized blunt needles were thrown out by single-use, disposable needles for acupuncture treatment currently, single-use, disposable cupping cups will be a market leader in future. Government policy can also promote this process as it is working in Korea.

## Figures and Tables

**Table 1:** The list of authorized companies producing disposable cupping cups which can be reimbursed by National Health Insurance Service in Korea.

Name	Materials	Product sizes (internal diameter, mm)	Website	First approval date
*SeongHo trade *	Polystyrene, silicone	50, 45, 37, 28, 22, and 15	http://www.9988.tv/	Jan. 1, 2012
*DongBang Acupuncture Inc. *	Polypropylene, silicone	49, 45, 37, 28, and 21	http://www.dbneedle.com/	Jan. 1, 2012
*LEADERS MEDITEC *	Polystyrene, silicone	53, 45, 29, and 22	Not available	Jan. 1, 2012
*DEmedical *	Polystyrene, plastic, and silicone	50, 40, 31, and 21	http://www.demedical.co.kr/	Feb. 1, 2012
*Haneng Lim Seo Won Medical *	Polystyrene	Unidentified	http://www.hlmedical.com/	Sep. 1, 2012
*Hihealth corp. *	Polystyrene	45, 39, 30, 25, and 20	http://www.hihealth.kr/	Sep. 1, 2012
*Goodpl *	Polyethylene, silicone	Unidentified	http://www.goodpl.kr/	Jan. 1, 2013
